# Supplementary material for: Nonlinear wave evolution with data-driven breaking
Source: Nat Commun. 2022 Apr 29;13:2343. doi: 10.1038/s41467-022-30025-z (PMC9054829; doi:10.1038/s41467-022-30025-z)
Supplement: Supplementary file 1 — Supplementary Information [file 41467_2022_30025_MOESM1_ESM.pdf]

Nonlinear wave evolution with data-driven breaking
Supplementary Information

SI 1 Limitations of infinitesimal-domain machine learning

In the infinitesimal-domain approach, the machine learning correction occurs at each numerical step. The machine learning correction is therefore detached from the nonlinear interactions that will mix the effects of the individual terms of the PDE after several solver steps and as such could give information about the physical processed captured in the RNN term. However, to apply this approach, the complex envelope of the true evolution is required at each model step to act as the initial condition for the MNLS solver step (see Figure 7b).

In our system, the phase information cannot be retrieved from experiments at the solver-step level. Figure SI 1.1 shows a comparison between an MNLS simulation and measurement data for a non-breaking wave. Let us define the complex envelope as $a(\xi,\tau) = |a(\xi,\tau)|^{i\phi(\xi,\tau)}$, that is, consisting of modulus $|a|$ and phase ϕ. Panel a and e demonstrate that the MNLS equation shows good agreement with the data for non-breaking waves. As the modulus of the envelope varies slowly with respect to the carrier wave, it is sufficiently sampled by the 12 gauges in the experiment, indicated by the green crosses in panels (c,g). Subsequently, one can obtain a smooth field at solver-step spacing through spline interpolation.

In contrast, the phase information is under-sampled with respect to the Nyquist frequency. This is indicated in fig. SI 1.1c,g by the red crosses corresponding to the data, and the red line corresponding to the MNLS simulation. We note that unwrapping the phase makes a direct comparison difficult. However, the discrepancy is highlighted in panel (d). The resulting trajectory in the phase plane (fig. SI 1.1d,h) for a given point in time is therefore significantly altered, and no unambiguous interpolation in the complex plane can be made. To our knowledge, it is therefore not possible to obtain the phase information at each solver step necessary to perform the infinitesimal-domain machine learning correction. In contrast, for the finite-domain machine learning (FDML) correction, Figure 7c, the phase information is only required for the initial condition.

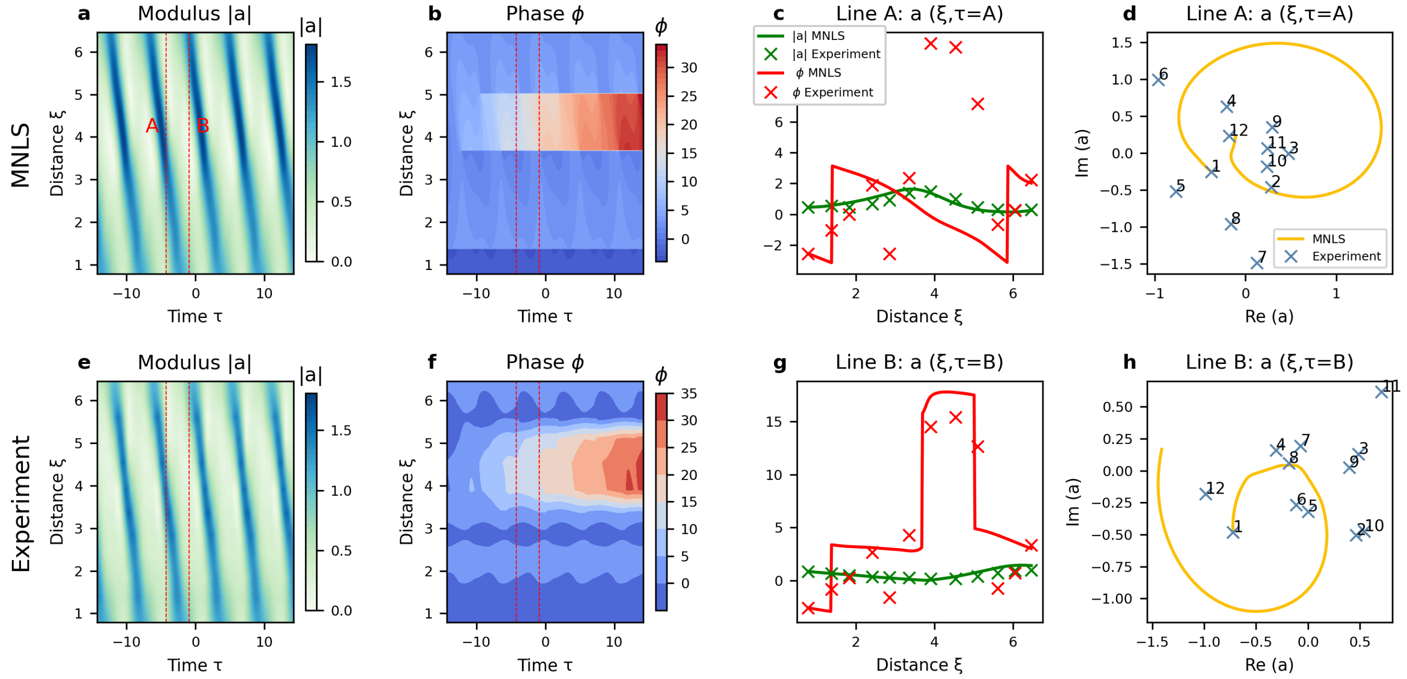

Figure SI 1.1: Modulus and phase information of complex envelope $a(\xi,\tau) = |a(\xi,\tau)|^{i\phi(\xi,\tau)}$. **a)** Modulus of the envelope, $|a|$, from MNLS simulations, and **e)** from measurements. **b)** Phase ϕ unwrapped in τ of the envelope from MNLS simulations, and **f)** from measurements. **c,g)** Modulus (green) and unwrapped phase (red) of MNLS simulations (solid line) and measurements (crosses) at cut-lines $\tau = A$ and $\tau = B$ (as shown in (a,e)), respectively. **d,h)** Phase trajectories in propagation direction ξ of the envelope at cut-lines $\tau = A$ and $\tau = B$ (as shown in (a,e)), respectively. For MNLS simulations (solid line) and measurements (crosses). The numbers of the crosses indicate the wave gauge position with respect to the wavemaker.

SI 2 Wave Category I: modulated plane waves

SI 2.1 Non-breaking wave example result

For a non-breaking wave, fig. SI 2.1 shows the MNLS+FDML framework only applies a slight correction to the MNLS, which already closely resembles the experiment.Note that while the MNLS offers a good approximation for a non-breaking wave, it does not include all the physics involved in the real experiment such as finite water depth, side wall friction, transverse modes and broad bandwidth. In contrast, the FDML correction is trained to learn the discrepancy between the measurement and the MNLS simulation, whether that is breaking or other inaccuracies. Therefore, it can outperform the MNLS also on non-breaking cases. Secondly, the FDML correction is optimized based on the MSE. However, especially in the time domain, the MSE is not a perfect metric, as it heavily penalizes small phase-shifts.

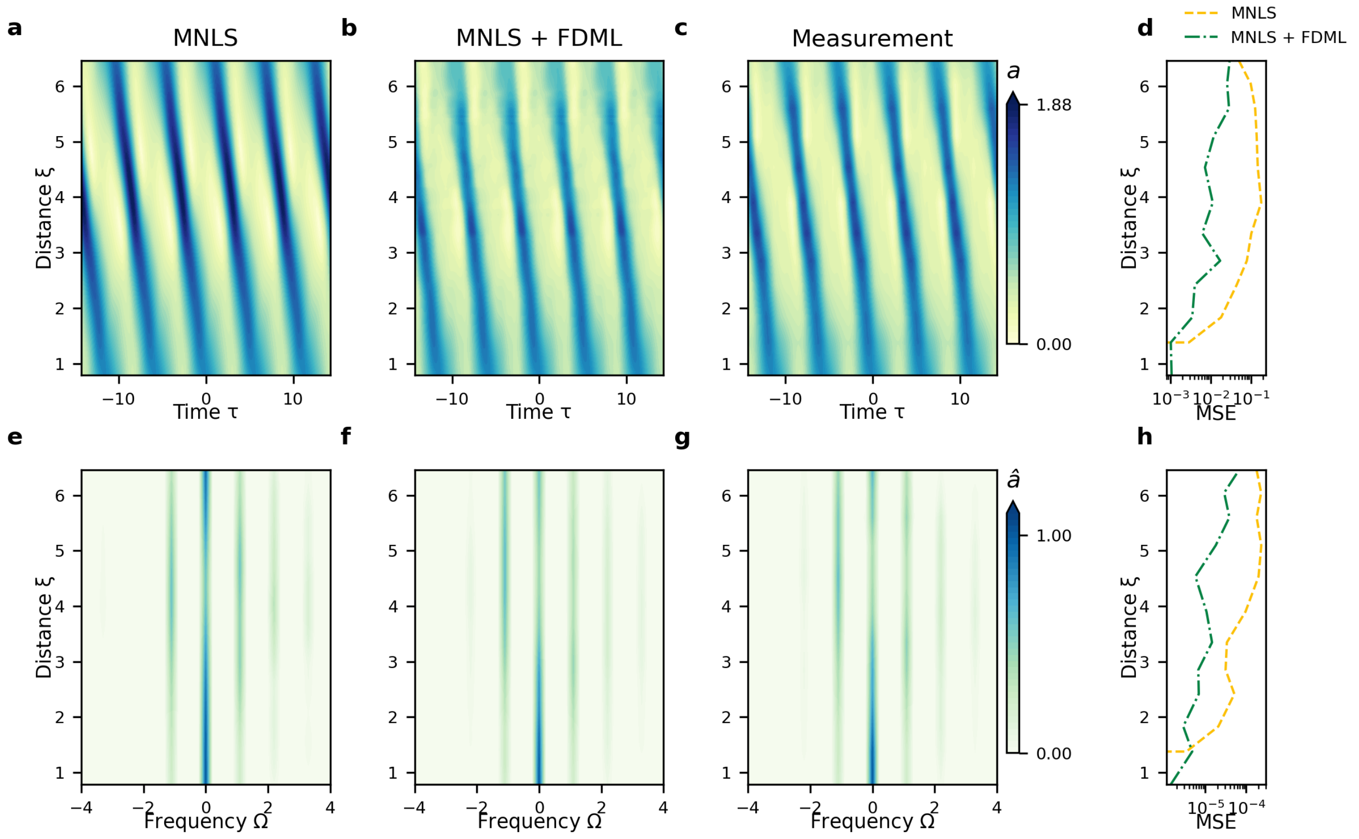

Figure SI 2.1: Example result (not used for training) of the spatial evolution of a non-breaking modulated plane wave (Wave Category I). **a-d)** Time domain. Color bar indicates envelope amplitude a (see eq. 1). **a)** MNLS simulations. **b)** MNLS+FDML simulations. **c)** Measurements. **d)** Mean squared error (MSE) at each wave gauge between measurements and MNLS and MNLS+FDML simulations. **e-h)** Frequency domain, similar panel configuration as in the time domain, with the color bar indicating the magnitude of the amplitude spectrum normalized by the maximum of the initial condition. Parameters of the initial conditions: $f_0 = 1.42$ Hz, $\tilde{a}_0 k_0 = 0.16$, $b_F = 0.16$, $\Omega_M = 1.1$, $\psi = 1.87\pi$ and $\alpha = 0$.

SI 2.2 Periodic boundary conditions example result

The MNLS is equivariant to translations in the time domain. Therefore, the FDML correction should also be able to apply corrections to arbitrarily translated input. This is achieved by including randomly translated inputs in the training set. Figure SI 2.2 shows the same example as in Figure 1, translated in τ by 60 time steps.

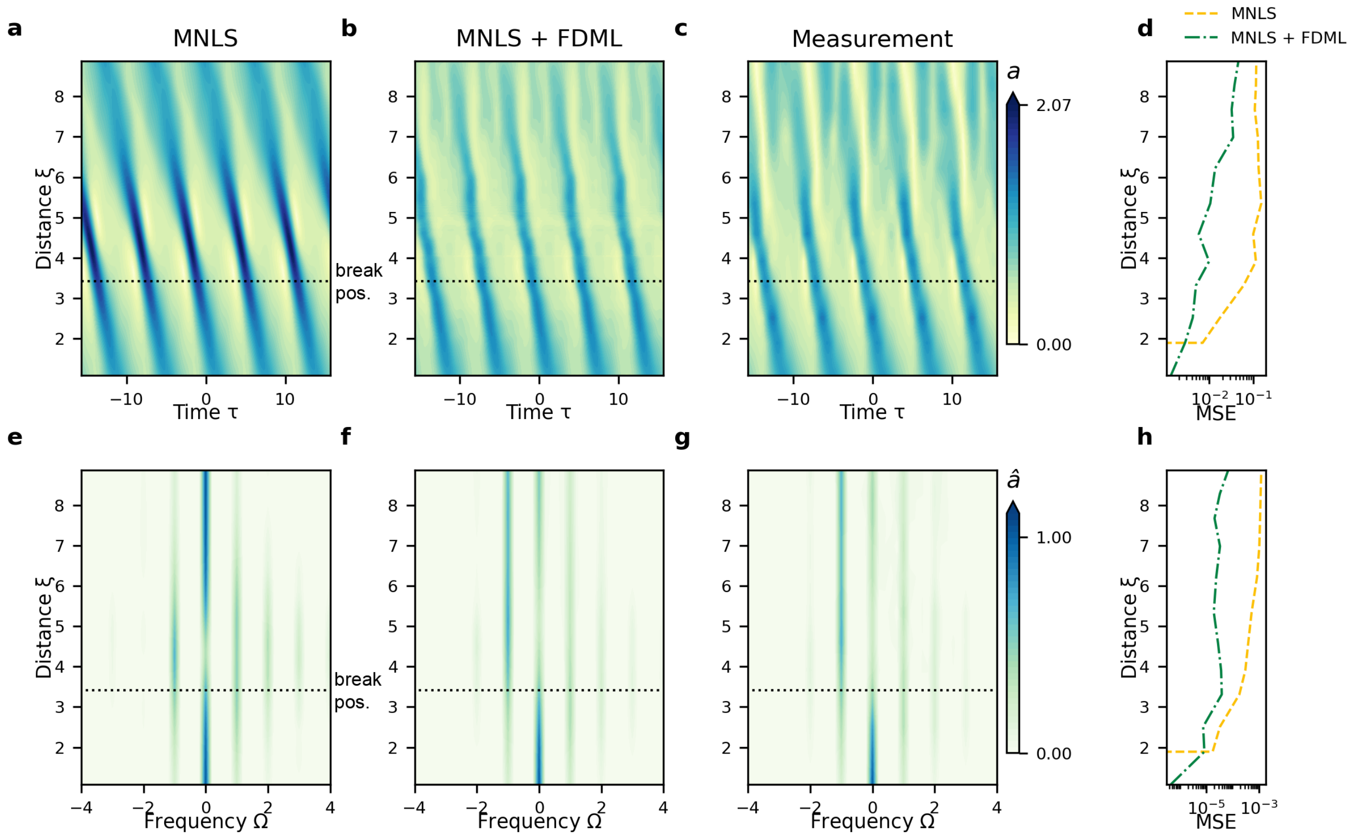

Figure SI 2.2: Example result (not used for training) of the spatial evolution of a breaking modulated plane wave translated in time (Wave Category I). **a-d)** Time domain. Color bar indicates envelope amplitude a (see eq. 1). **a)** MNLS simulations. **b)** MNLS+FDML simulations. **c)** Measurements. **d)** Mean squared error (MSE) at each wave gauge between measurements and MNLS and MNLS+FDML simulations. **e-h)** Frequency domain, similar panel configuration as in the time domain, with the color bar indicating the magnitude of the amplitude spectrum normalized by the maximum of the initial condition. Parameters of the initial conditions are same as in Figure 1.

SI 2.3 Experiments in the test set

Figures SI 2.3 and SI 2.4 show the experiments from the test set for Wave Category I (summarized in Figure 5). Note that these are for evolution over the whole length of the tank, starting from the first wave gauge. Augmenting the data by starting at different points and using smaller propagation lengths could create many more samples.

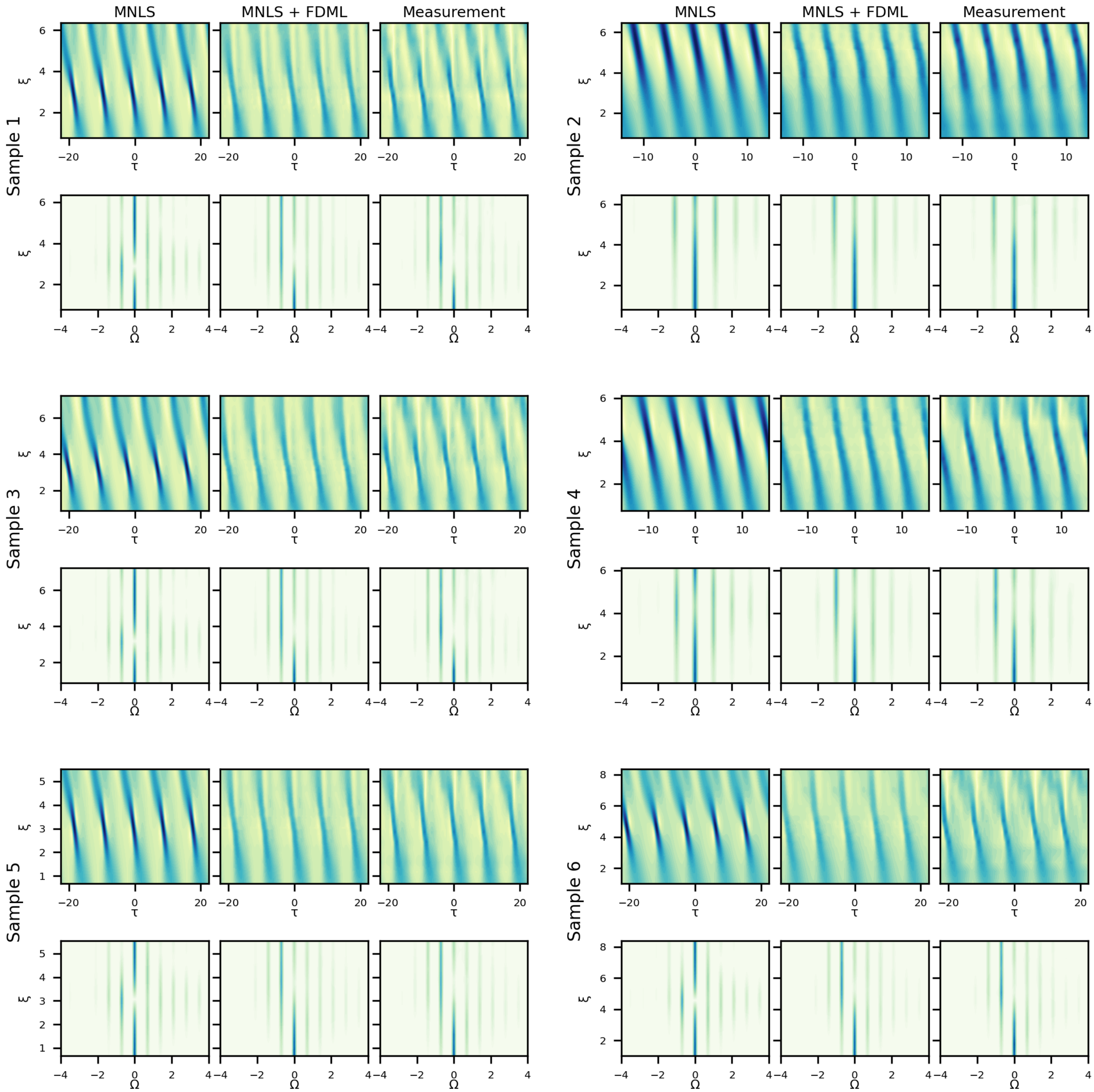

Figure SI 2.3: Test experiments 1-6 in Wave Category I. Each with a different combination of wave parameters.

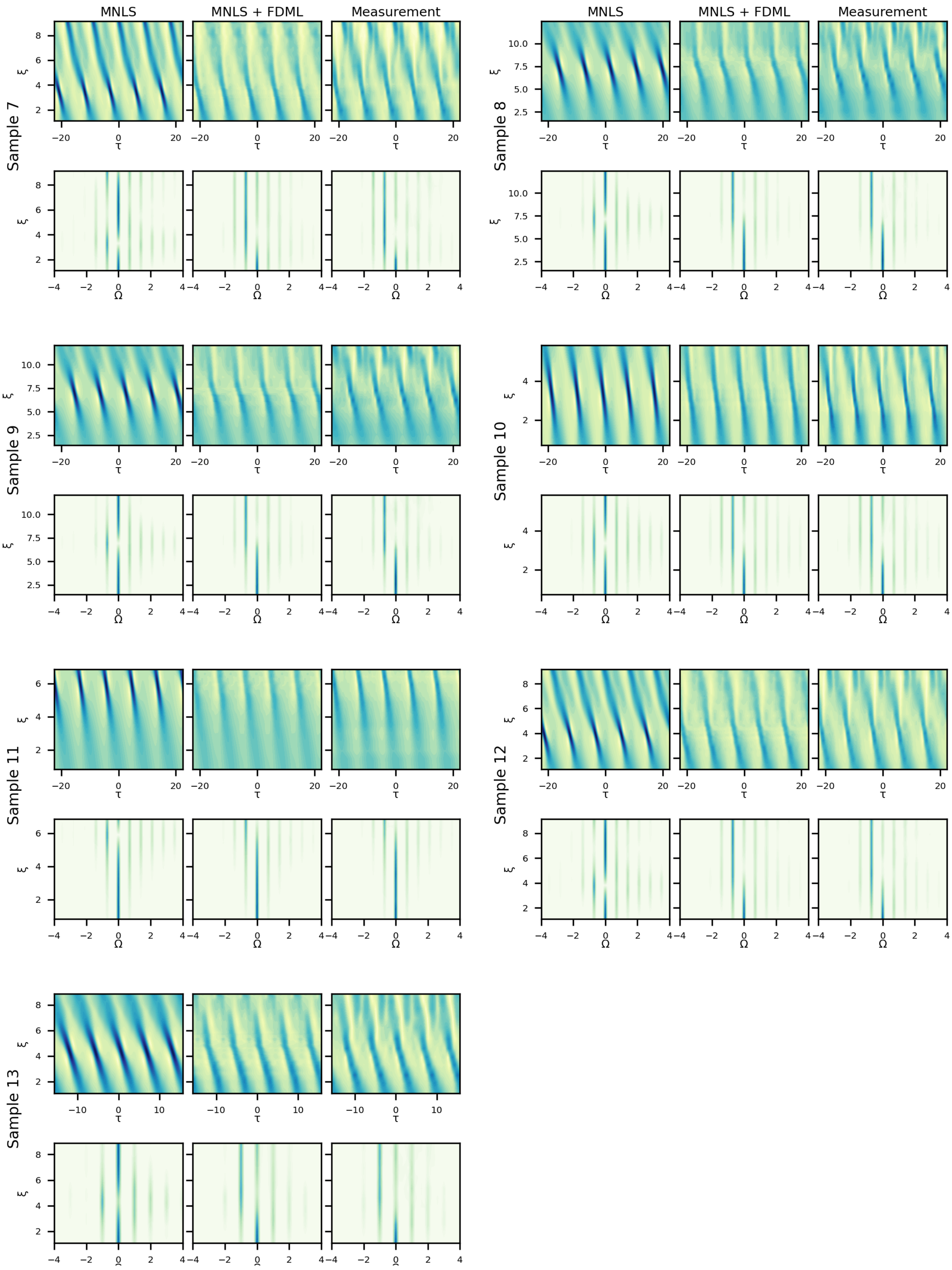

Figure SI 2.4: Test experiments 7-13 in Wave Category I. Each with a different combination of wave parameters.

SI 3 Wave Category II: dispersively focused irregular waves

SI 3.1 Non-breaking wave example result

The evolution of a focused packet that does not undergo wave breaking confirms the FDML correction does not apply a significant correction in the non-breaking case. In fig. SI 3.1 the MNLS and MNLS+FDML prediction are nearly identical in the frequency domain; thus no correction is applied, as confirmed by the low and comparable MSE (fig. SI 3.1h). In physical space, the FDML correction applies a slight over-damping.

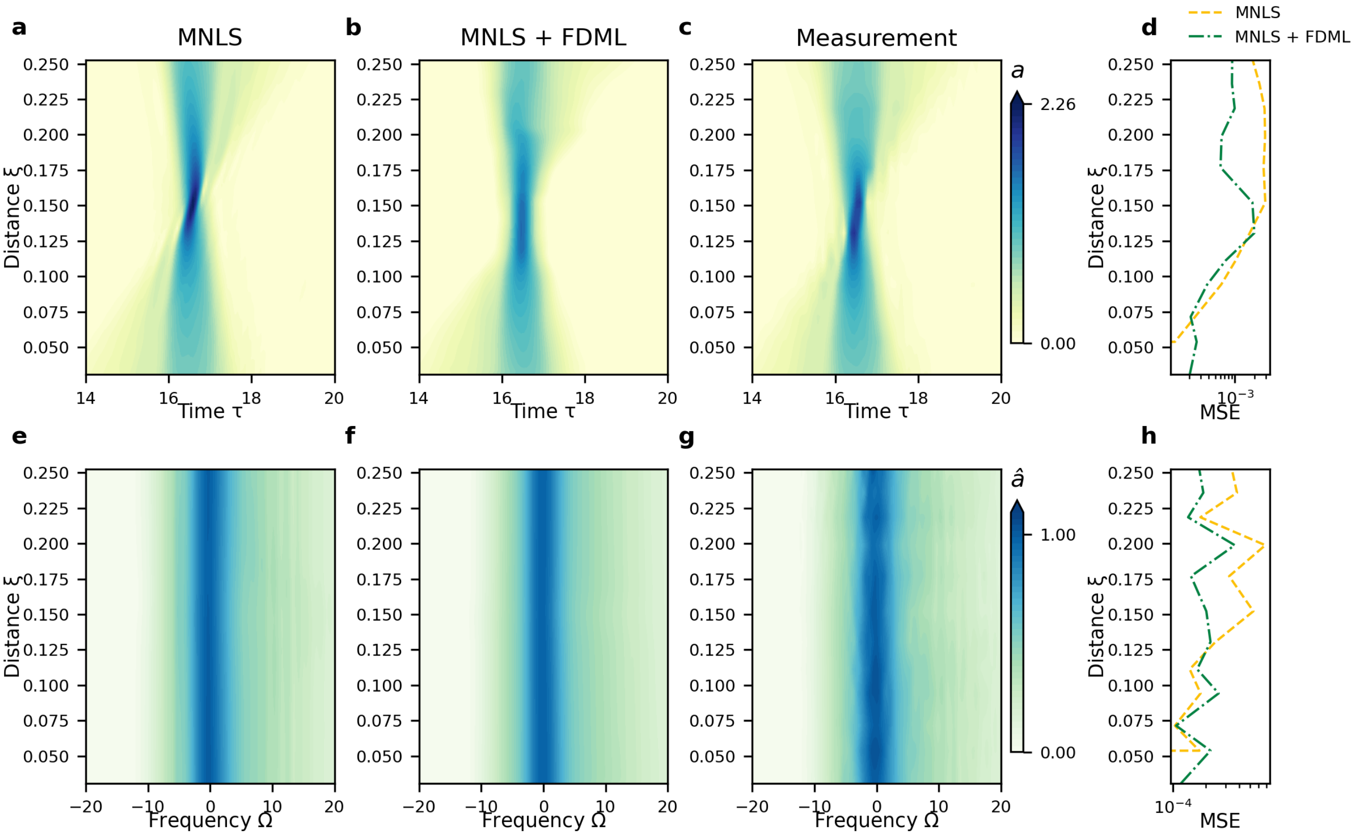

Figure SI 3.1: Example result (not used for training) for the spatial evolution of non-breaking dispersively focused irregular waves (Wave Category II). **a-d)** Time domain. Color bar indicates elevation envelope a. **a)** MNLS simulations. **b)** MNLS+FDML simulations. **c)** Measurements. **d)** Mean squared error (MSE) at each wave gauge between measured envelope and MNLS and MNLS+FDML. **e-h)** Frequency domain, similar panel configuration as in the time domain, with the color bar indicating the magnitude of the spectrum normalized by the maximum of the initial condition. Parameters of the initial conditions: $f_p = 0.91$ Hz, ($\omega_p = 5.7$ rad/s), $\gamma = 3.3$, $H_s = 10$ mm and $x_f = 18$ m.

SI 3.2 Example result with different starting point and propagation length

The MNLS+FDML solver can start from any arbitrary initial condition, as long as the complex envelope is available. In fig. SI 3.2 the initial condition is the third wave gauge in the tank. The propagation length of the simulation can be set arbitrarily. In fig. SI 3.2, the propagation length is set such that the simulation extends beyond the physical tank length, indicated by the white space in panels c and g. Wave parameters are the same as in Figures 2-4.

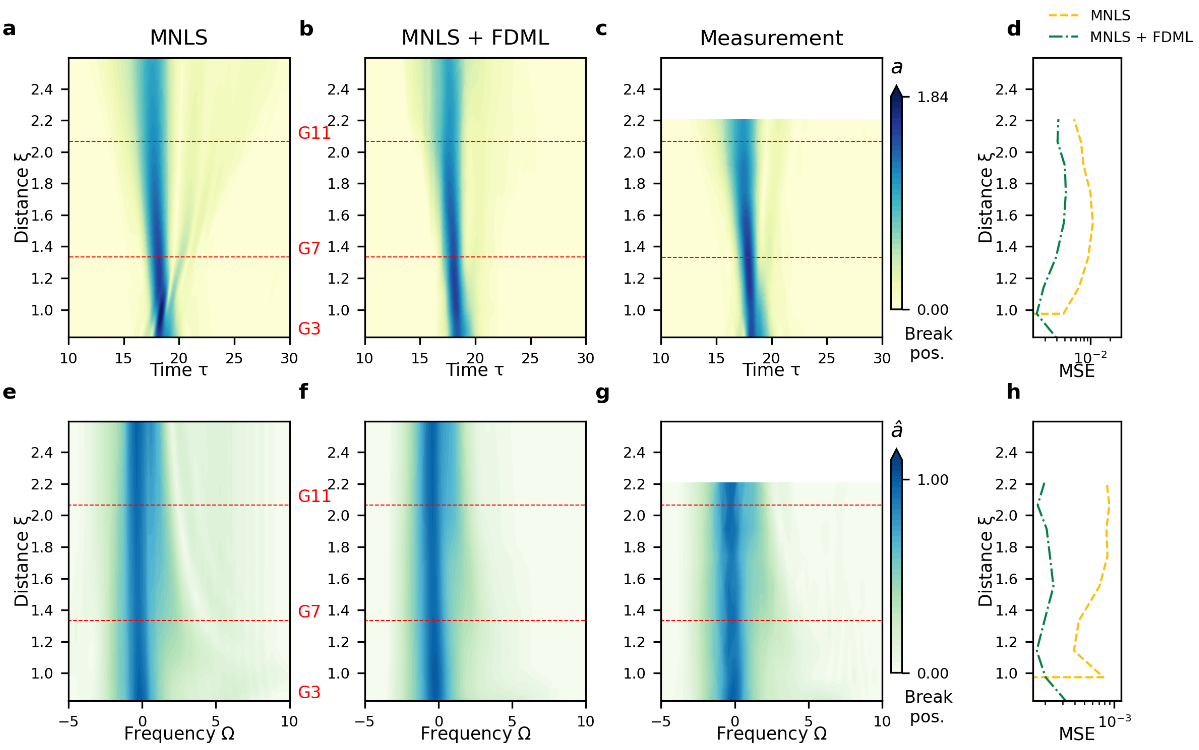

Figure SI 3.2: Example result (not used for training) for the spatial evolution of dispersively focused irregular waves (Wave Category II) with alternative starting point and propagation length. **a-d)** Time domain. Color bar indicates elevation envelope a. **a)** MNLS simulations. **b)** MNLS+FDML simulations. **c)** Measurements. **d)** Mean squared error (MSE) at each wave gauge between measured envelope and MNLS and MNLS+FDML. **e-h)** Frequency domain, similar panel configuration as in the time domain, with the color bar indicating the magnitude of the spectrum normalized by the maximum of the initial condition. Parameters of the initial conditions are the same as in Figures 2-4.

SI 3.3 Experiments in the test set

Figures SI 3.3 and SI 3.4 show the experiments from the test set of Wave Category II (summarized in Figure 5). Note that these for evolution over the whole length of the tank, starting from the first wave gauge. Augmenting the data by starting at different points and using smaller propagation lengths could create many more samples.

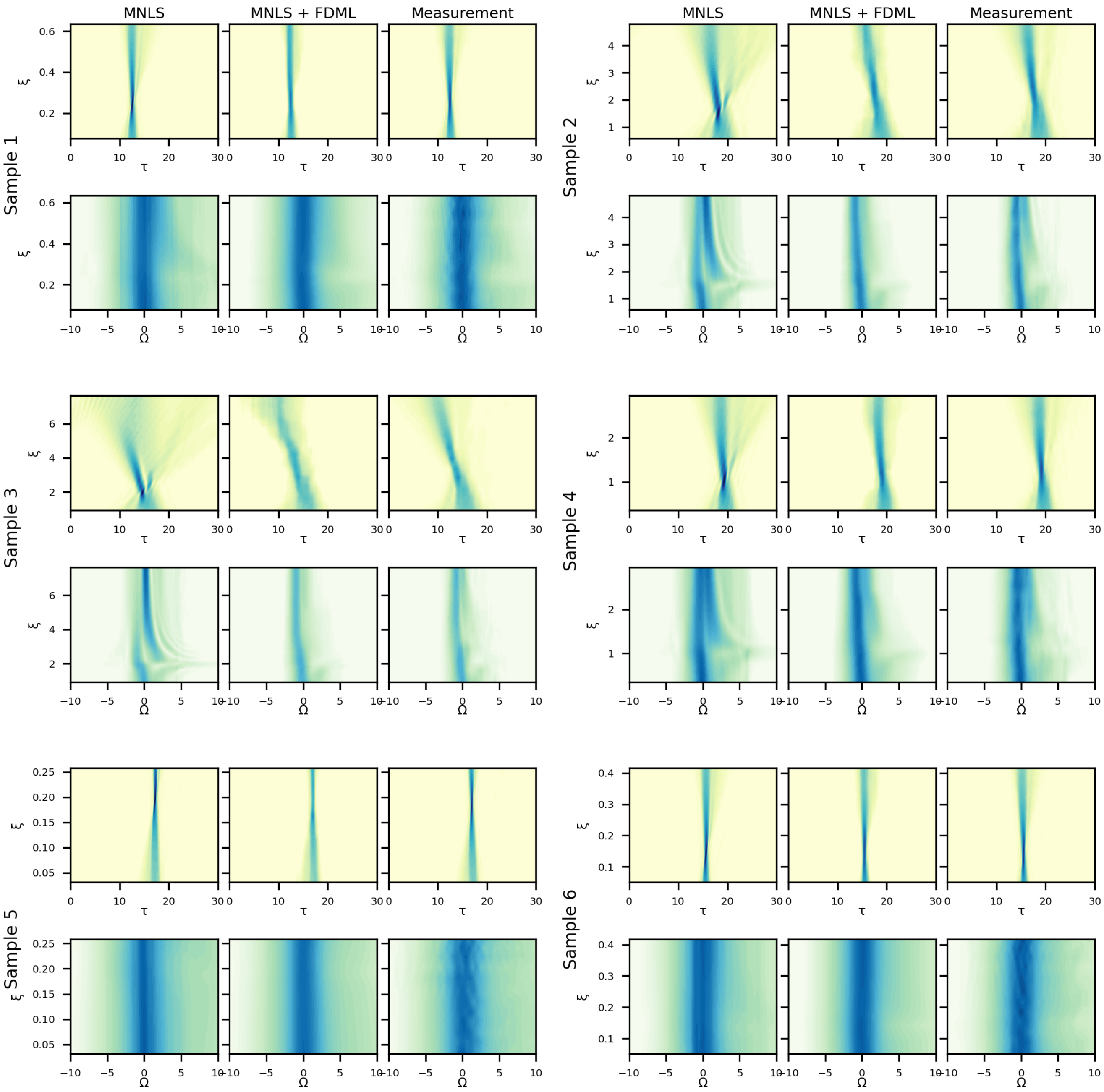

Figure SI 3.3: Test experiments 7-13 in Wave Category II. Each with a different combination of wave parameters.

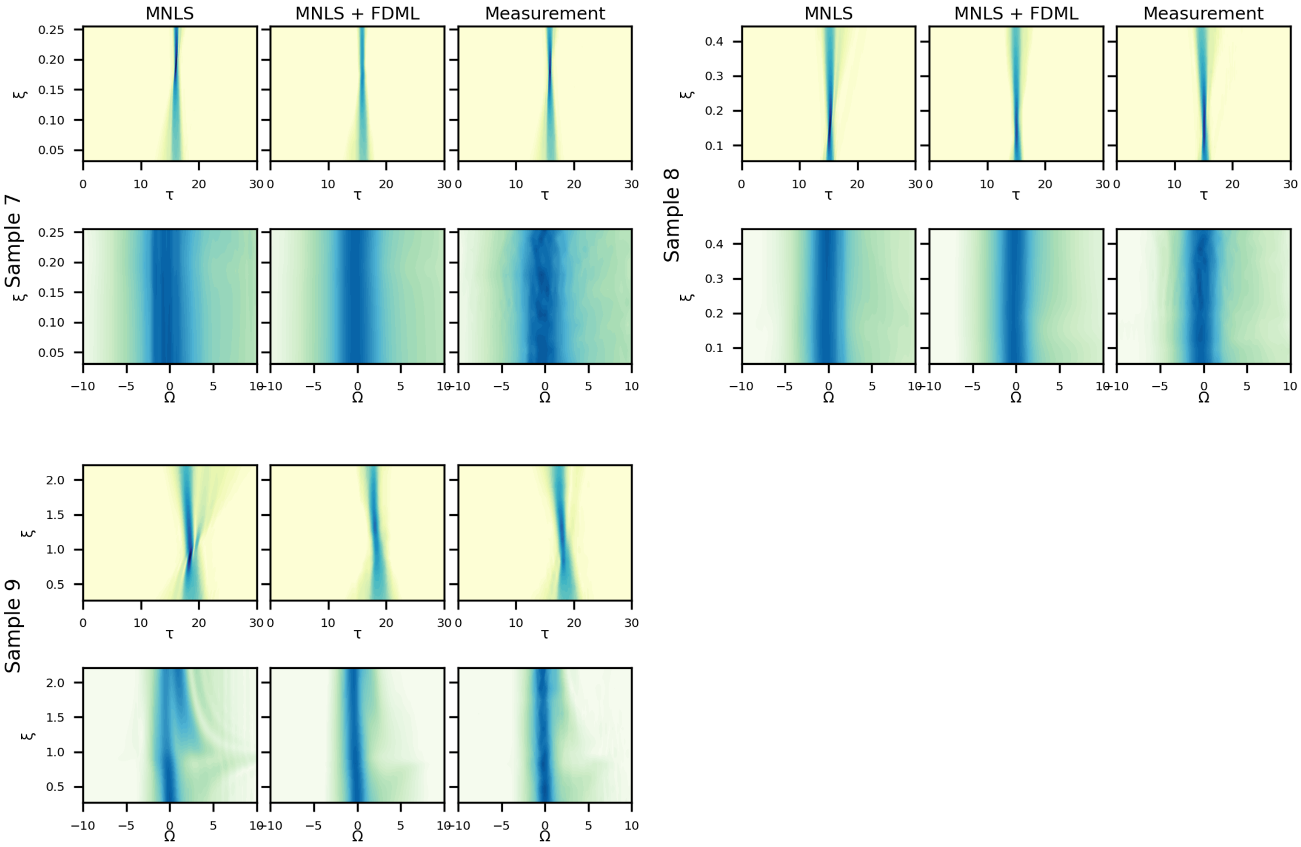

Figure SI 3.4: Test experiments 7-13 in Wave Category II. Each with a different combination of wave parameters.

SI 3.4 External dataset

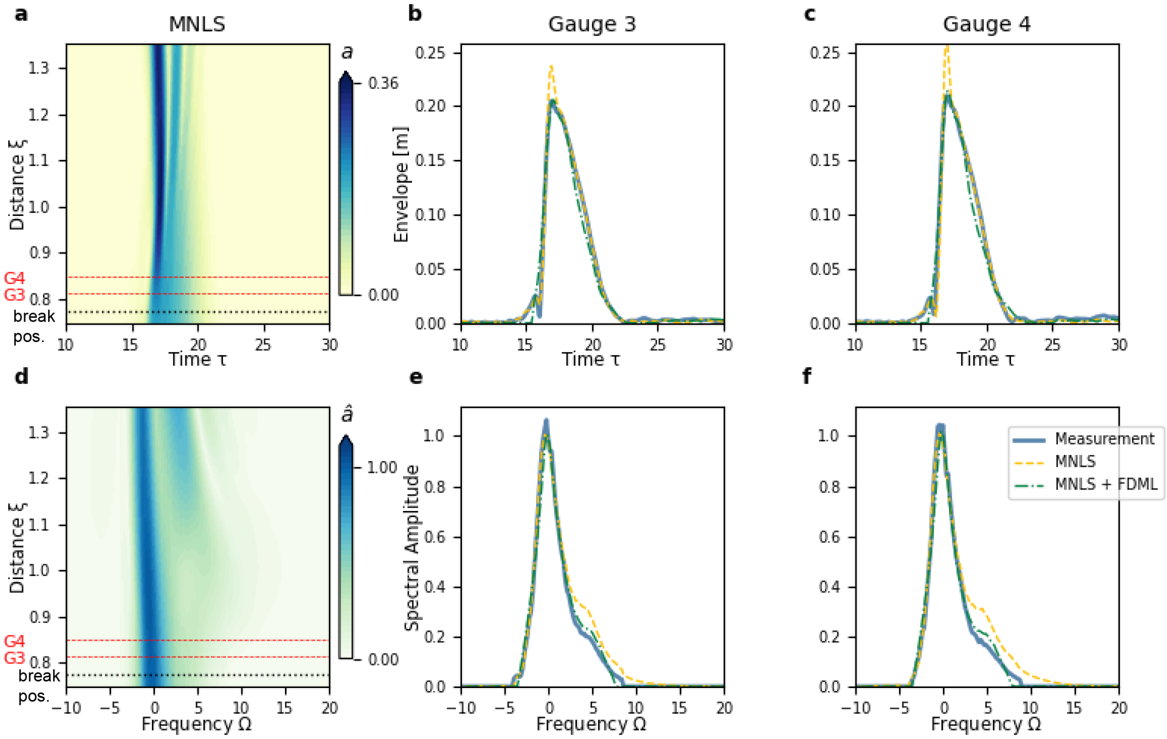

Figure SI 3.5: Verification of the MNLS+FDML model on an external data set obtained at Shanghai Jiao Tong University (not used for training) with breaking occurring at $\xi = 0.77$ ($x = 76$ m). **a-c)** Time domain. **d-f)** Spectrum. **a,d)** MNLS simulation. **b,e)** Gauge 3 **c,f)** Gauge 4.

To verify the generalization properties and robustness of our MNLS+FDML model, we use it to predict wave breaking in an external data set of focused breaking waves recorded in the Multifunctional Ship Model Towing Tank at Shanghai Jiao Tong University. This tank used to has a length of 300 m, a width of 16 m, and a water depth of 7.3 m. Initial parameters were based on a Gaussian spectrum with $f_p = 0.47$ Hz ($\omega_p = 2.95$ rad/s) variance 0.06, $H_s = 120$ mm and linear focusing at $x_f = 144$ m. The initially Gaussian spectrum was focused based on linear wave theory to reach a maximum amplitude at $\xi_f = 1.46$ from the wavemaker, but broke at $\xi \ = \ 0.77$ ($x = 76$ m). Four wave gauges are placed along the centre-line of the tank at ξ =0.75, 0.78, 0.81 and 0.85. Breaking occurs between the first and second gauge. Due to the non-linearity of the waves, the spectrum broadens as the waves evolve, and a spectral tail develops, giving the spectrum a more JONSWAP-like shape. The initial condition for the simulations is the surface elevation record from the first wave gauge.

Figure SI 3.5a,d, displaying the physical space and spectral evolution respectively, show that the wave gauges only record a very small part of the total focusing-defocusing cycle of the wave, while the MNLS+FDML model was trained to cover larger parts of the cycle. Nevertheless, the FDML correctly suppresses the shoulder in the spectrum upon breaking (fig. SI 3.5e,f) and caps off the high peak of the envelope predicted by the MNLS (fig. SI 3.5b,c).

The significant wave height of this wave group was 120 mm, corresponding to a wave amplitude-based Reynolds number [1] of $\mathrm{Re} = a_s^2\omega_p/\nu = 1.1 \times 10^4$, where $a_s = H_s/2$ is the significant amplitude. This is a much larger wave than in the experiments used to train the network. For the example in Figures 2-4, $\mathrm{Re} = 1.3 \times 10^3$ ($H_s = 30$mm), an order of magnitude lower. This demonstrates the robustness of our MNLS+FDML model across a range of scales.

SI 4 Comparison with Kato & Oikawa (1995)

SI 4.1 Wave Category I: modulated plane waves

For modulated plane waves (Wave Category I), the wave breaking model developed in Kato & Oikawa (1995) [2] is able to reproduce some features of wave breaking, such as energy dissipation, amplitude attenuation and shortening of the FPUT recurrence cycle. However, it fails to capture the full strength of the downshift in the spectrum, and therefore gives different dynamics at greater distance from the breaking point. The KO95 model has one free parameter β (see their equation 2.1). When β is set too low there is no effect on the evolution. When set too high there is too much dissipation and the wave field disappears. For fig. SI 4.1, $\beta = 0.2$ was set such that the effect on the evolution was maximal, while the amount of energy dissipation remained reasonable.

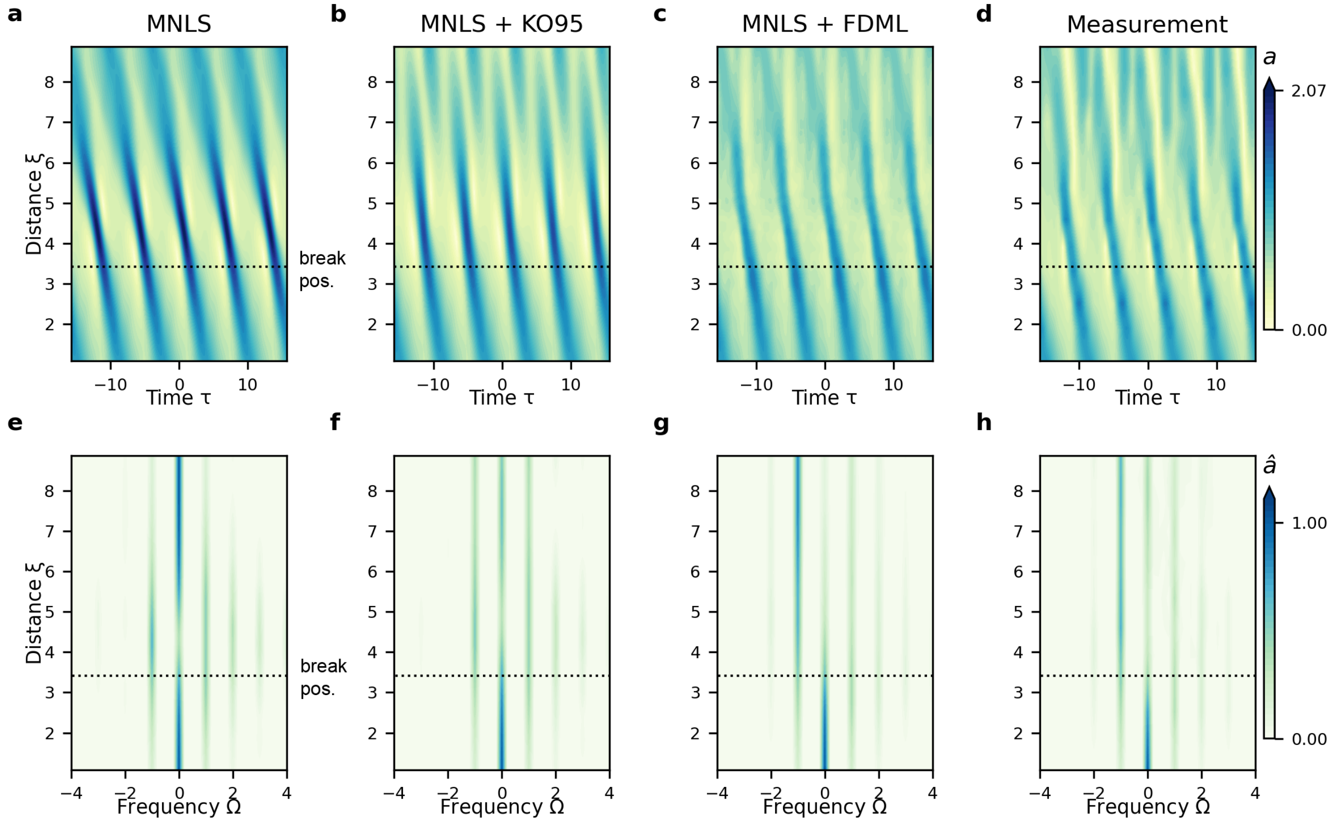

Figure SI 4.1: Comparison to the breaking model of Kato & Oikawa (1995) (KO95) for the evolution of a breaking modulated plane wave (Wave Category I). **a-d)** Time domain. Color bar indicates elevation envelope a. **a)** MNLS simulations. **b)** MNLS+KO95 simulations. **c)** MNLS+FDML simulations. **d)** Measurements. **e-h)** Frequency domain, similar panel configuration as in the time domain, with the color bar indicating the magnitude of the spectrum normalized by the maximum of the initial condition. Parameters of the initial conditions are the same as in Figure 1

.

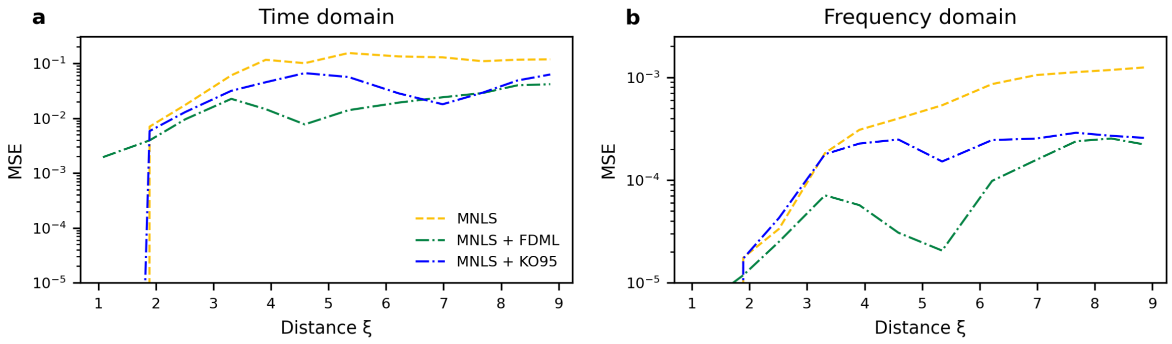

Figure SI 4.2: Mean squared error (MSE) at each wave gauge between measured envelope and MNLS, MNLS+FDML and MNLS+KO95 corresponding to fig. SI 4.1. **a)** Time domain. **a)** Frequency domain.

.

SI 4.2 Wave Category II: dispersively focused irregular waves

For the dispersively focused irregular waves (Wave Category II), the KO95 [2] wave breaking model was performed with $\beta = 0.3$ (see their equation 2.1). Like for Wave Category II, this parameter was tuned such that it would have a maximal effect, limited by a reasonable dissipation of the energy of the wave. The MNLS + KO95 model gives a slight attenuation of the peak amplitude, but seems to turn on too late (fig. SI 4.3b). That is, after the breaking event in the experiment has already taken place. Suppression of the higher frequencies is only very slight in the frequency domain (fig. SI 4.3f). Overall, for the continuous spectrum of Wave Category II, the KO95 correction term is not as effective as for Wave Category I. The modification to the MNLS evolution is only minimal, as reflected by their similar MSE values in fig. SI 4.4.

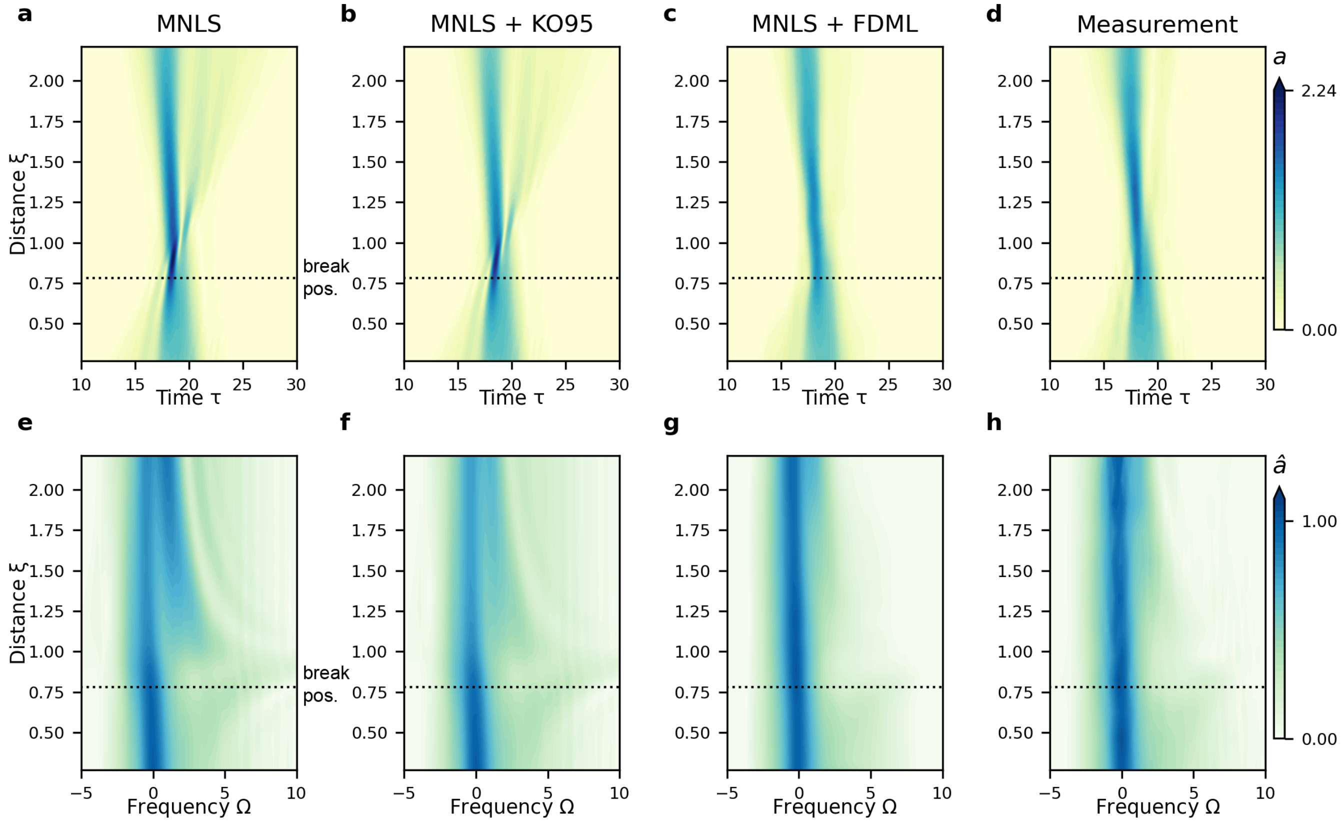

Figure SI 4.3: Comparison to the breaking model of Kato & Oikawa (1995) (KO95) for the evolution of breaking dispersively focused irregular waves (Wave Category II). **a-d)** Time domain. Color bar indicates elevation envelope a. **a)** MNLS simulations. **b)** Kato 1995 simulations. **c)** MNLS+FDML simulations. **d)** Measurements. **e-h)** Frequency domain, similar panel configuration as in the time domain, with the color bar indicating the magnitude of the spectrum normalized by the maximum of the initial condition. Parameters of the initial conditions are the same as in Figures 2-4.

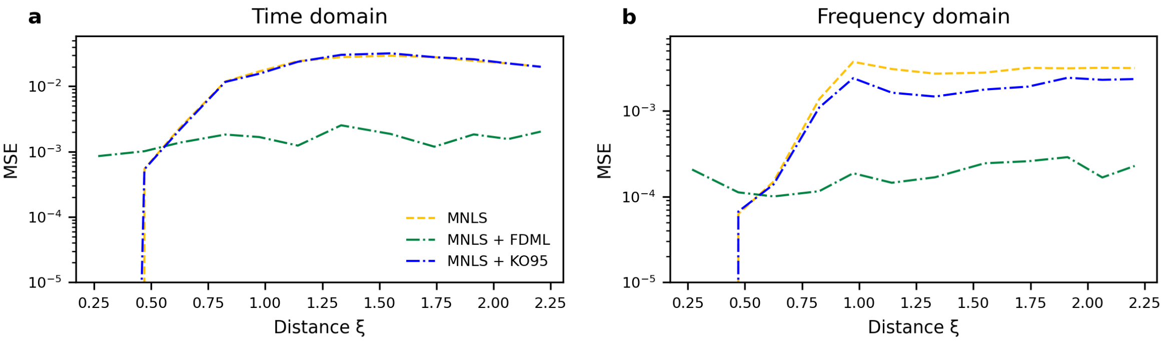

Figure SI 4.4: Mean squared error (MSE) at each wave gauge between measured envelope and MNLS, MNLS+FDML and MNLS+KO95 corresponding to fig. SI 4.3. **a)** Time domain. **a)** Frequency domain.

.

SI 5 Wave Category III: random irregular waves

SI 5.1 Training data and algorithm

The goal for Wave Category III is to be able to predict the evolution of a finite time series up to a certain prediction horizon.The available experimental data consists of three time series of 20 minutes, or 1500 wave periods, for four different wave heights: $H_s = 25, 34, 41, 54$ mm, with a total of 12 experiments. For the lowest wave height there are no breaking events, for the highest value many breaking events occur.

Training pairs are created as follows. After determining a time and spatial step, the envelope is interpolated to those steps over the domain. Then, sections of 512 time steps and either 60 or 120 propagation steps are created, as illustrated in fig. SI 5.1a by the dashed rectangles. For each wave gauge, the next section is shifted 64 steps from the previous, as illustrated by the red rectangle, shifted from the yellow. This is repeated for each subsequent wave gauge. The green rectangle is positioned at the second wave gauge.

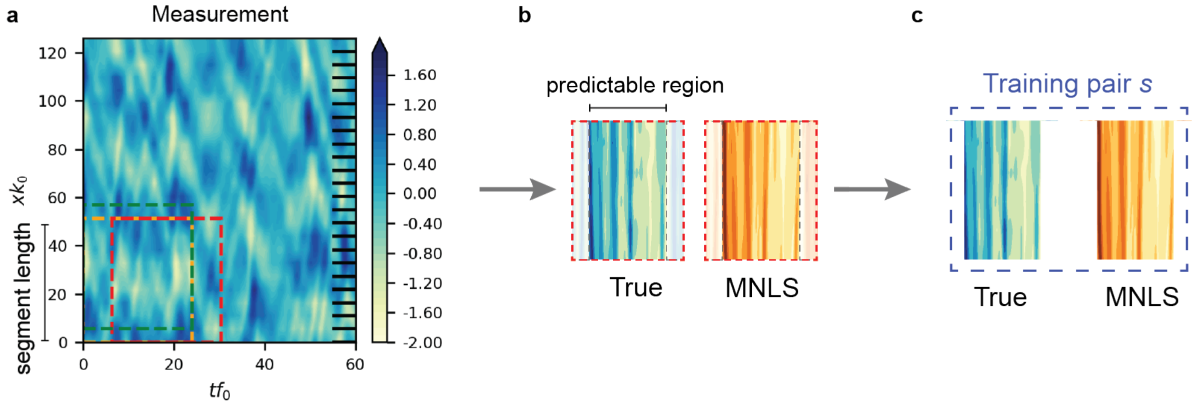

Figure SI 5.1: **a)** Sections in space and time are created from the total wave record for random irregular waves (Wave Category III). **b)** For each section, an MNLS simulation is performed. **c)** For both the simulation and the measurement, the edges are discarded to account for information getting lost near the edges in the simulation to create a truncated section. A training pair s consists of the MNLS simulation and the interpolated measurement over the section.

For each section created, the boundaries of the first time step are made periodic such that it can serve as an initial condition for the MNLS simulation. The MNLS simulation is performed over the segment length (fig. SI 5.1b). To take into account the fact that information can leave and come in through the boundaries, we discard the outer 64 time steps of both the measurement and the MNLS simulation, in analogue to the principle of the predictable region. This reduces the number of time steps from 512 to 386 (fig. SI 5.1b). Note that to be rigorous, this predictable region should depend on the propagation length, and the spectral properties, but we ignore this here. These truncated sections then form a training pair s, as shown in fig. SI 5.1c. To account for the truncated input and the amount of information contained in this input, the network architecture is slightly different than for the other wave categories. It consists of 384 input neurons, 384 output neurons, 256 LSTM neurons, and 256 dense neurons. The rest of the training algorithm is identical to that of Wave Categories I and II, outlined in the Methods section and SI 6.

For each of the 12 experiments, a section in time is reserved for validation (grey shaded) and testing (white shaded), as marked shaded areas in fig. SI 5.2. These areas will not be used for training.

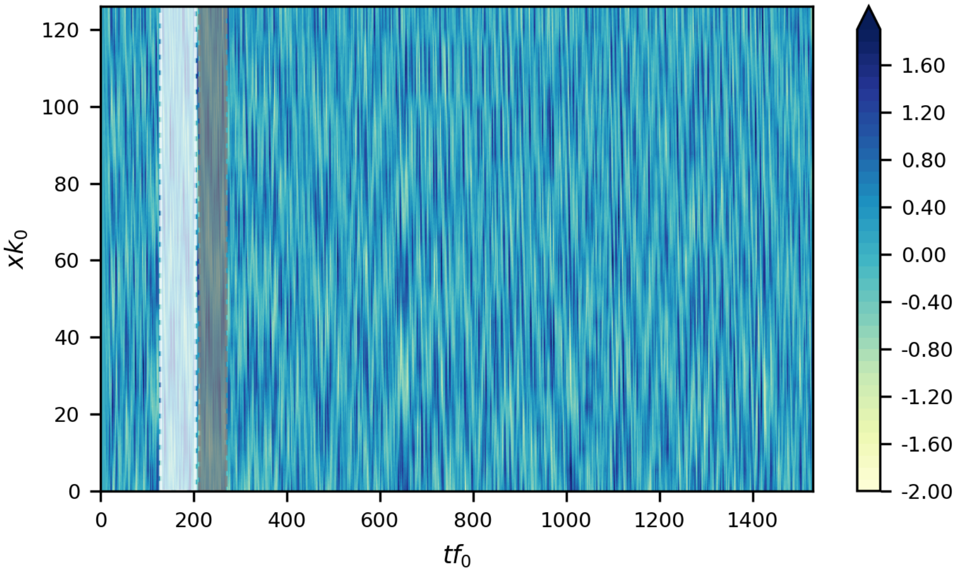

Figure SI 5.2: Example of a total experiment for random irregular waves (Wave Category III). Test-section is colored white, validation section is colored grey.

SI 5.2 Narrow-banded result: $\gamma = 6$

Note the clear reduction in the MNLS+FDML model MSE at the wave breaking location. Also note that the model was trained on about 1/5 of the propagation length displayed here. Due to the narrower spectrum, the predictable region is extended.

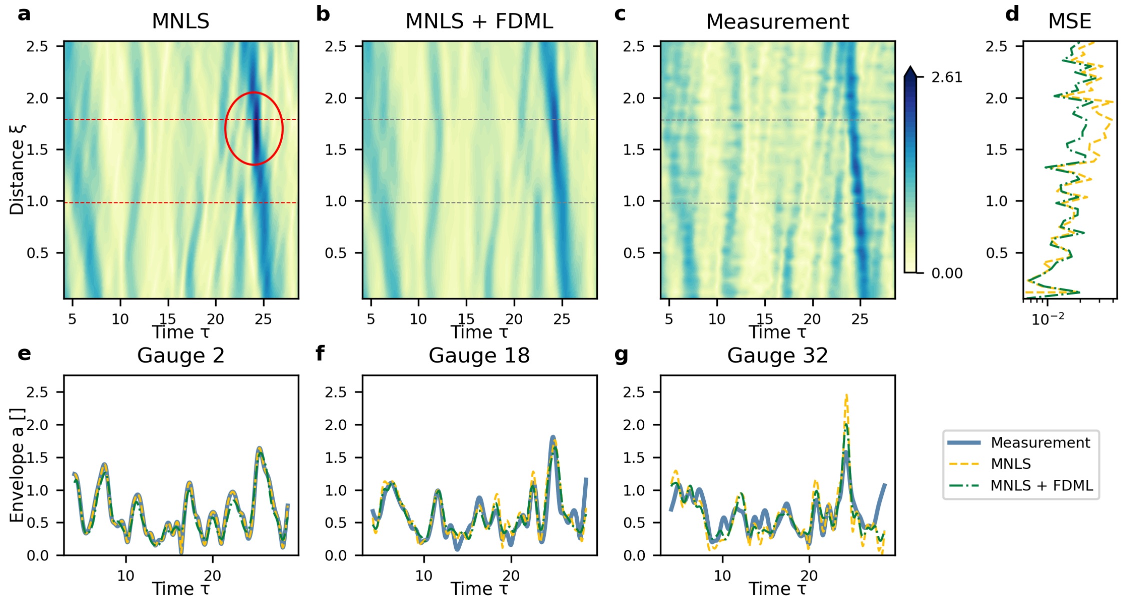

Figure SI 5.3: Example result (not used for training) for the evolution of the envelope irregular waves (Wave Category III), for a narrow-banded sea: $\gamma = 6$, in the time domain, showing wave breaking at $\xi = 1.7$, as indicated by the red circle. Color bar indicates surface elevation envelope a. **a)** MNLS simulations. **b)** MNLS+FDML simulations. **c)** Measurements. **d)** Mean squared error (MSE) at each wave gauge between measured envelope and MNLS and MNLS+FDML. **e-g)** Envelope at different spatial locations: measurements (solid blue), MNLS simulations (dashed yellow) and MNLS+FDML simulations (dashed-dotted green) at the wave gauge locations indicated by the red-dotted lines in (a). The red circle indicates a wave breaking event.

SI 5.3 Other wave examples

As a clear breaking signature is difficult to identify in an irregular sea, we identify steep waves instead, which may or may not break, and illustrate the performance of the MNLS+FDML method in the vicinity of these steep events. Figure SI 5.4 shows in red the regions of steepness $\tilde{a}k_0 > 0.28$, for each of the three cases with $H_s = 0.54$ mm (the steepest experiment). Each possible breaking event is indicated with a number. Figures SI 5.5 and SI 5.6 show the MNLS and MNLS+FDML simulation for each event.

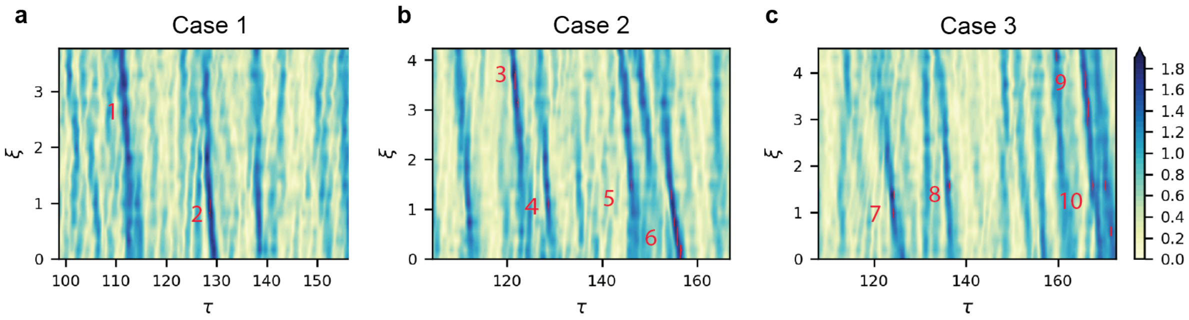

Figure SI 5.4: Test region for the three instances of the $H_s = 54$ mm experiment. Regions with steepness $\tilde{a}k_0 > 0.28$ are marked in red and numbered.

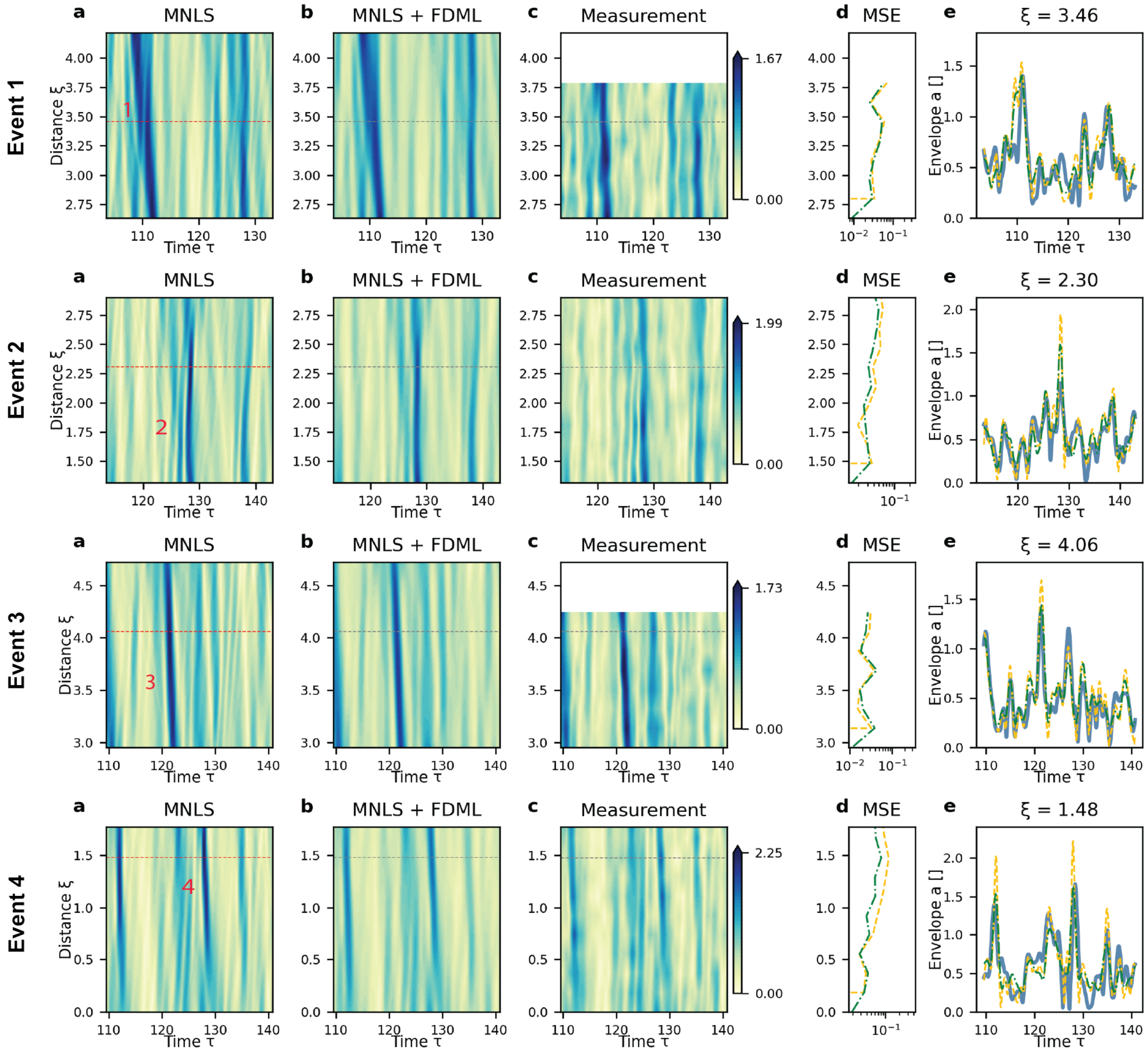

Figure SI 5.5: Simulations around the regions indicated in fig. SI 5.4

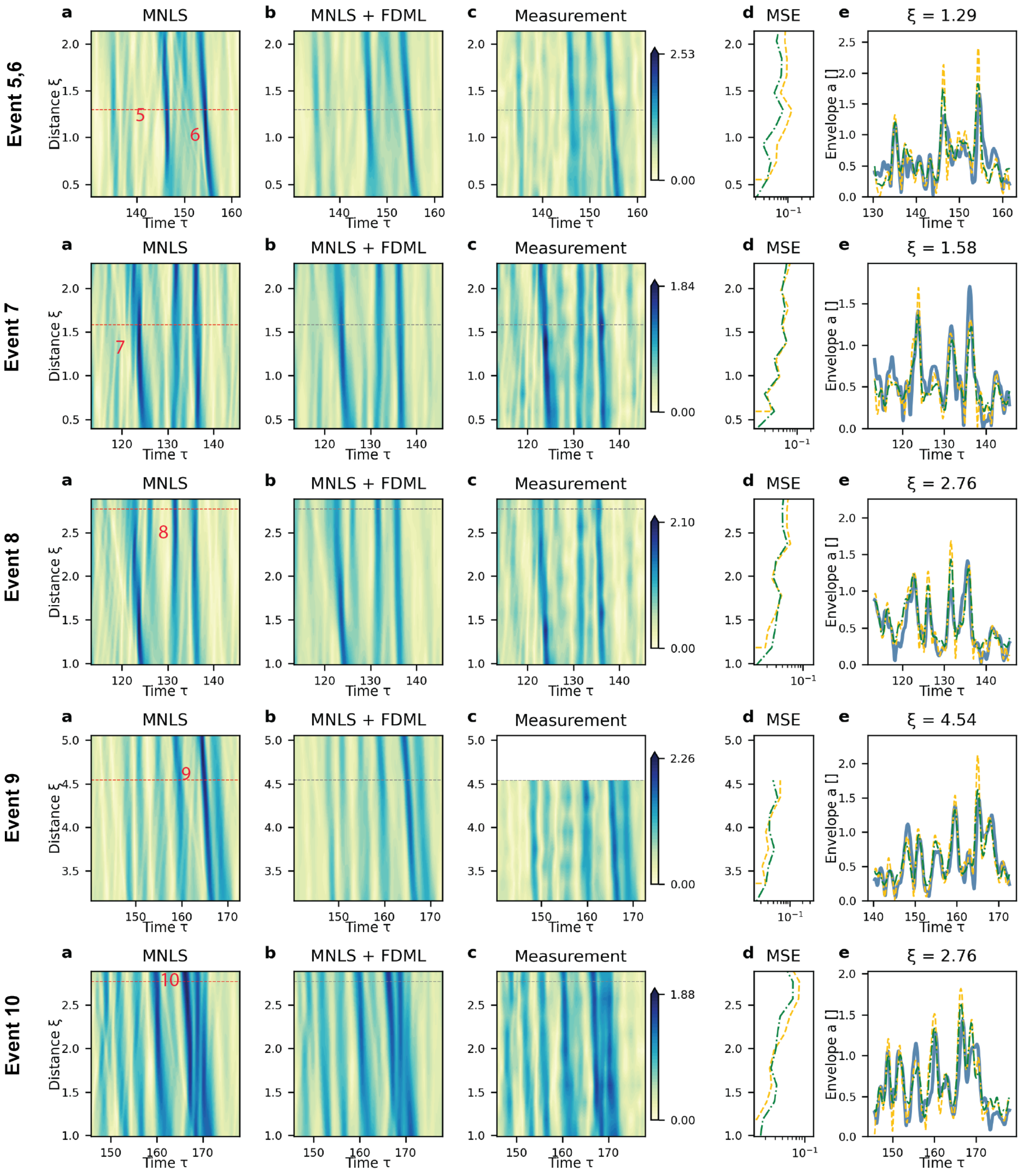

Figure SI 5.6: Simulations around the regions indicated in fig. SI 5.4

SI 5.4 MSE test set

Figure SI 5.7 displays the MSE averaged over both space and time for the first 200 data pairs from the test-set (as in fig. SI 5.1c, but for the test region), of each wave height, the dashed line indicating the MNLS+FDML model, and the solid line the MNLS model. While figures Figure 6 and fig. SI 5.3 show a local dip in MSE at the location of a wave breaking event, this does not affect the global MSE of the sample. Indeed, the performance of the MNLS and the MNLS+FDML models are comparable in fig. SI 5.7, indicating that averaged MSE is not a useful metric to confirm the ability of the MNLS+FDML model to predict breaking in irregular seas, in which breaking remains sporadic.

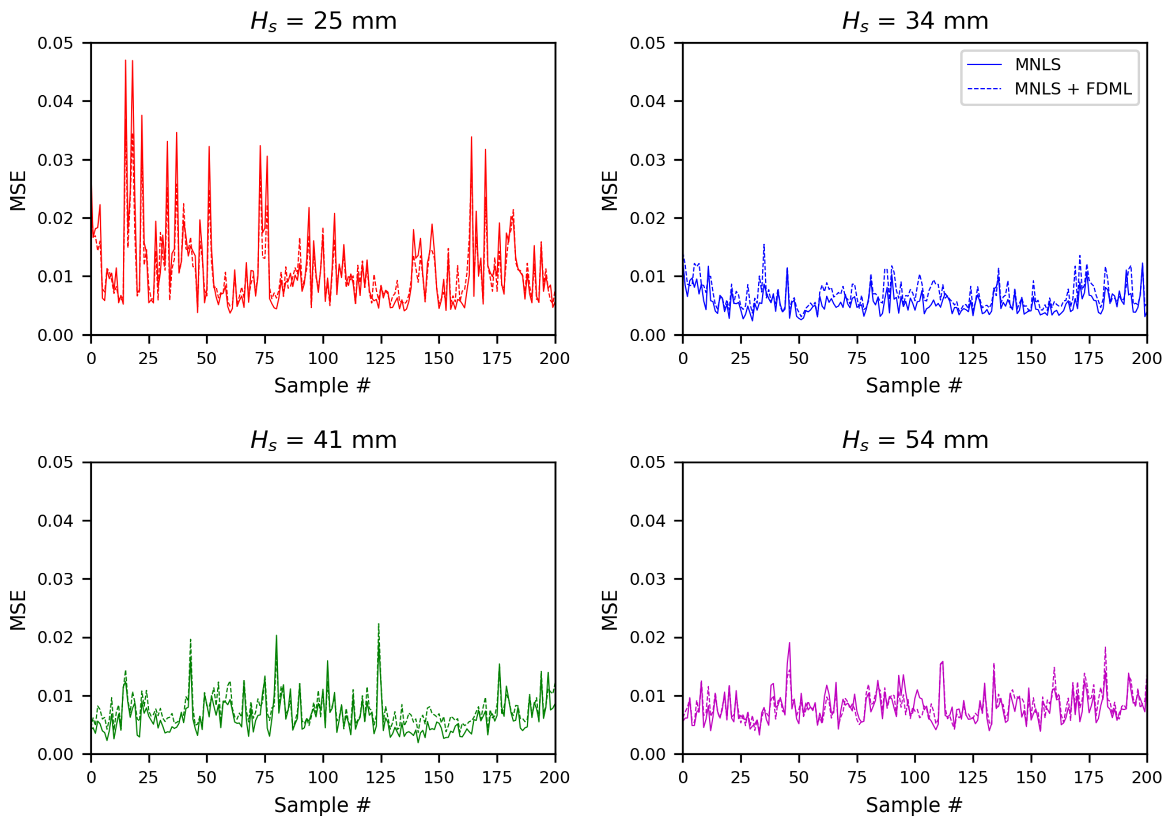

Figure SI 5.7: Mean Squared Error (MSE) for the first 200 test samples of each wave height. **a)** $H_s = 25$ mm **b)** $H_s = 34$ mm **c)** $H_s = 41$ mm **d)** $H_s = 44$ mm.

SI 6 Neural network and algorithm

The three algorithms (Algorithm 1-3) below outline the training procedure of the neural network for Wave Categories I and II. The method for Wave Category III is slightly different, as the raw data is a long continuous record rather than separate experiments, and is outlined in SI 5.

SI 6.1 Algorithm 1: Obtain ground truth at solver step

The wave tank experiments need to be interpolated such that the ground truth is available at every solver step, instead of only at the wave gauge positions. In both the time and the frequency domain, the absolute value of the envelope varies slowly in the propagation direction and is sufficiently sampled according to the Nyquist–Shannon theorem, and can be interpolated using a *pchip* spline. To make the algorithm applicable to different data-sets, the propagation and time-step are scaled so that the amount of variation over a typical length scale are comparable. The length of the time vector corresponds to the number of input neurons of the network and should therefore be high enough such that the MNLS can be solved, but not too high to avoid too many parameters to be tuned. In our study we used $n_t = n_{\text{RNN}} = 512$ for wave Categories I and II, and $n_t = n_{\text{RNN}} = 384$ for wave Category III . Note that in the illustration of algorithm 1, we show the evolution of only one of these time-points.

Algorithm 1: Obtain ground truth at solver step

---

procedure GROUND TRUTH($\mathcal{E}$)
  $\mathcal{E}\{e_1, e_2, ...e_{\text{N}_\text{E}}\}$ ▷*Set of experiments, based on N_E unique wave parameter combinations*
  Set $\Delta\xi_{\text{train}} = k_0 \Delta x = 0.2$ ▷*Determine scaled solver step*
  Set $\Delta\tau_{\text{train}} = \omega_0 \epsilon \Delta t = 0.01$ ▷*Determine scaled time step*
  for $e \in \mathcal{E}$ **do** ▷*Loop over experiments*
    $\Delta x = 0.2/k_0$
    $\Delta t = 0.01/(\omega_0 \epsilon)$
    for PS,FS **do**: ▷*PS = Physical space, FS = Fourier space*
      $e[\Delta t_{\text{wave gauge}}, \Delta x_{\text{wave gauge}}] \longrightarrow \bar{e}[\Delta t, \Delta x]$ ▷*Interpolate*
    end for
    $\bar{\mathcal{E}}\{\bar{e}_1, \bar{e}_2, ...\bar{e}_{\text{N}_\text{E}}\}$ ▷*Set of interpolated experiments*
  end for
end procedure

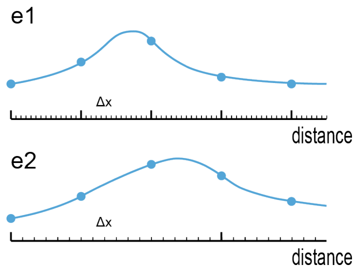

SI 6.2 Algorithm 2: Data augmentation and creation of training pairs

To increase the number of learning cases, each experiment is split up in segments of different propagation lengths and with different starting points, as shown in fig. SI 6.1. For each experiment, 3 segments lengths are selected. Only segments terminating before the end of the wave tank, i.e., segments that are fully covered by the experiments are used for training. The starting position can only be at wave gauge positions, as these are the only locations with access to the complex envelope (modulus + phase).

Algorithm 2: Algorithm 3. MNLS simulations for training pairs

procedure MNLS SIMULATIONS FOR TRAINING PAIRS($\bar{\mathcal{E}}$)
    for $\bar{e} \in \bar{\mathcal{E}}$ **do**
        $n_{\text{steps,seg}} = l_i$, $i = 1, 2, 3$ ▷ *Chose 3 different segment lengths*
        for l_i **do**: k=1 ▷ *Gauge number k*
            while $x_k + l_i \leq x_{max}$ **do** ▷ *While the segment fits in the tank*
                $a_{\text{true}}(0, l_i) = \bar{e}(x_k, x_k + l_i)$ ▷ *Select segment of true propagation*
                $a_{\text{MNLS,0}} = a_{\text{true,0}}$ ▷ *Get complex initial condition from wave gauge*
                $j = 1$
                while $j \leq n_{\text{steps,seg}}$ **do**
                    $a_{\text{MNLS,j}} = \text{MNLS}\ (a_{\text{MNLS,j-1}})$ ▷ *Propagate MNLS solver over segment using split-step Fourier method*
                    $j = j + 1$
                end while
                for PS,FS **do**: ▷ *Save training pair in PS and FS*
                    save: $s = \{|a|_{\text{MNLS}}[n_{\text{steps,seg}}, 512], |a|_{\text{true}}[n_{\text{steps,seg}}, 512]\}$
                end for
            end while
        end for
    end for
    $\mathcal{S}\{s_1, s_2, ...s_{\text{N}_\text{S}}\}$ ▷ *Set of training pairs, based on N_S unique wave parameter-, segment length- and start position combinations*
end procedure

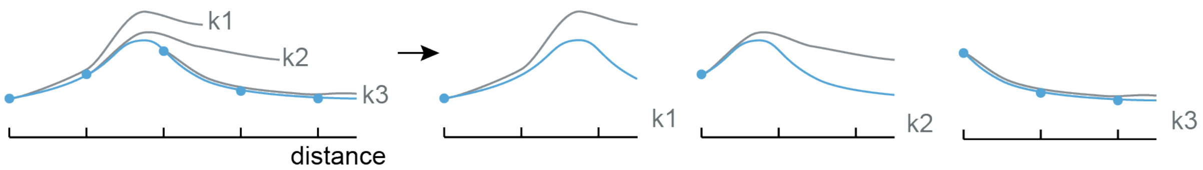

Figure SI 6.1: Different starting point give different discrepancies between MNLS and true evolution.

SI 6.3 Algorithm 3. Network architecture and training

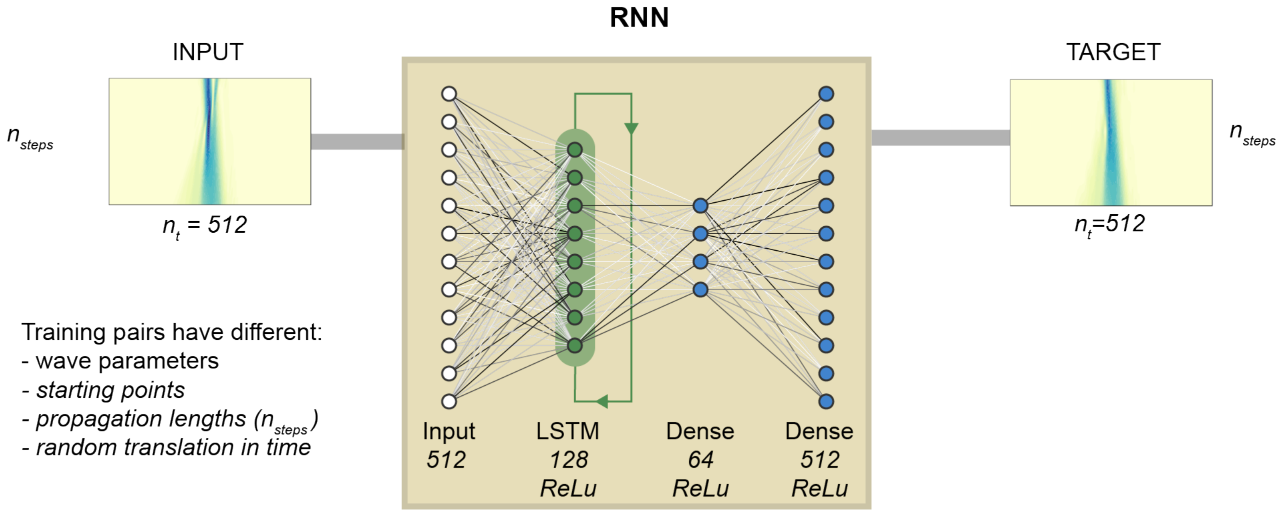

Figure SI 6.2: Network architecture

As described in the Methods section, the network architecture is a simple LSTM, depicted in fig. SI 6.2. The parameter vector of the network β consists of all the weights and biases of each neuron, as well as additional parameters for the gate functions of the LSTM unit. The goal is find the optimal β such that the cost function, eq. 10 is minimized. To this end, the network is fed with training pairs $s = [\text{input}, \text{output}] = [|a|_{\text{MNLS}}(\xi_k, \xi_k + n_{\text{steps}}\Delta\xi), |a|_{\text{true}}(\xi_k, \xi_k + n_{\text{steps}}\Delta\xi)]$ in batches. To minimize eq. 1 over each batch, several optimization schemes can be employed. We use the *Adam* gradient descent variant provided by the TensorFlow package, to which we have added a gradient limit to avoid explosion of the gradient. The training procedure is outlined in Algorithm 3. As the MNLS is equivariant to translations of the time axis and has periodic boundary conditions, in the time domain, 40 random translations in time are added to augment the data (i.e., to create more training pairs).

Algorithm 3: Training procedure

procedure TRAINING($\mathcal{W}, \bar{\mathcal{E}}$)
  $\mathcal{E}\{e_1, e_2, ...e_{\text{N}_\text{E}}\}$ ▷*Set of experiments, based on N_E unique wave parameter combinations*
  $\mathcal{S}\{1, 2, ...n_\text{S}\}$ ▷*Set of training pairs, based on N_S unique wave parameter-, segment length (l)- and start position (x_0) combinations*
  Shuffle $\bar{\mathcal{E}}$
  $\textbf{fraction}_{\text{train}} = 0.80$, $\textbf{fraction}_{\text{val}} = 0.15$, $\textbf{fraction}_{\text{test}} = 0.05$
  $\longrightarrow \bar{\mathcal{E}}_{\text{train}}, \bar{\mathcal{E}}_{\text{val}}, \bar{\mathcal{E}}_{\text{test}} \subset \bar{\mathcal{E}}$
  $\longrightarrow \mathcal{S}_{\text{train}}, \mathcal{S}_{\text{val}}, \mathcal{S}_{\text{test}} \subset \mathcal{S}$ ▷*Obtain corresponding segment length and start position combinations*
  for PS,FS **do**: ▷*PS = Physical space, FS = Fourier space*
    RNN(β_0, a) ▷*Initialize RNN with random parameters β_0*
    for n_{epochs} **do** ▷*Optimize using 'Adam' gradient descent*
      for n_{batches} **do**
        $l_i \in L$ ▷*Randomly draw a segment length* [1]
        $s \in \mathcal{S}_{\text{train}} = \{|a|_{\text{MNLS,i}}[n_{\text{steps}}, 512], |a|_{\text{true,i}}[n_{\text{steps}}, 512]\}$ ▷*Input, Output pair of training sample i*
        Calculate J (eq. 10)
      end for
      Minimize $J(\beta)$ (eq. 10) $\longrightarrow \beta$ ▷*Minimize cost function, update parameters*
      Print: MSE training and MSE validation
    end for
  end for
end procedure

[1]Note that an LSTM can only be trained on batches of equal length

SI 6.4 Using the MNLS-FDML solver

The utilization of the MNLS-FDML scheme is the same as that of the MNLS solver, but also includes breaking effects, i.e., it takes the form of an initial value problem or deterministic wave forecasting. That is, for a given initial condition at a position x_0 we would like to know the evolution up to and including a point x_1. If the initial condition is a time series measurement of the surface elevation, the complex envelope $a(x_0, t)$ has to be obtained using the Hilbert transform (see main text). Subsequently, the MNLS solver can be used to propagate the solution forward an arbitrary number of steps. After this propagation, the RNN applies a correction for the absolute value of the physical envelope and its Fourier spectrum, as depicted in Figure 7d.

Algorithm 4: Utilization MNLS-FDML correction

procedure MNLS-FDMLC($\eta_{x_0,t}$)
  given: $\eta_{x_0,t}$ ▷*Given a time series measurement at position x_0*
  $\eta_{x_0,t} \longrightarrow A_{x_0,t}$ ▷*Obtain complex envelope*
  $A_{x_0,t} \longrightarrow a_{\xi=0,\tau}$ ▷*Make Nondimensional*
  Set $\Delta\xi = 0.2$ ▷*Set time and propagation step solver*
  Set $\Delta\tau = 0.01$
  Set n_{steps} ▷*Set desired number of propagation steps*
  while $i \leq n_{\text{steps}}$ **do**
    ▷ Propagate MNLS solver over desired length using the Split-Step scheme
    $a_{\text{MNLS,i}} = \text{MNLS}\ (a_{\text{MNLS,i-1}})$
    $i = i + 1$
  end while
  for PS,FS **do**: ▷*Apply correction over propagated steps*
    $|a|_{\text{pred}} = \mathbf{RNN}(\beta, |a|_{\text{MNLS}})$
  end for
end procedure

SI 7 - Training History

Figure SI 7.1 shows the mean squared error (MSE) of the training and validation set as a function of training epoch. In one epoch, the neural network is trained with all the training data once. The training data is then shuffled to continue training for the next cycle or epoch.

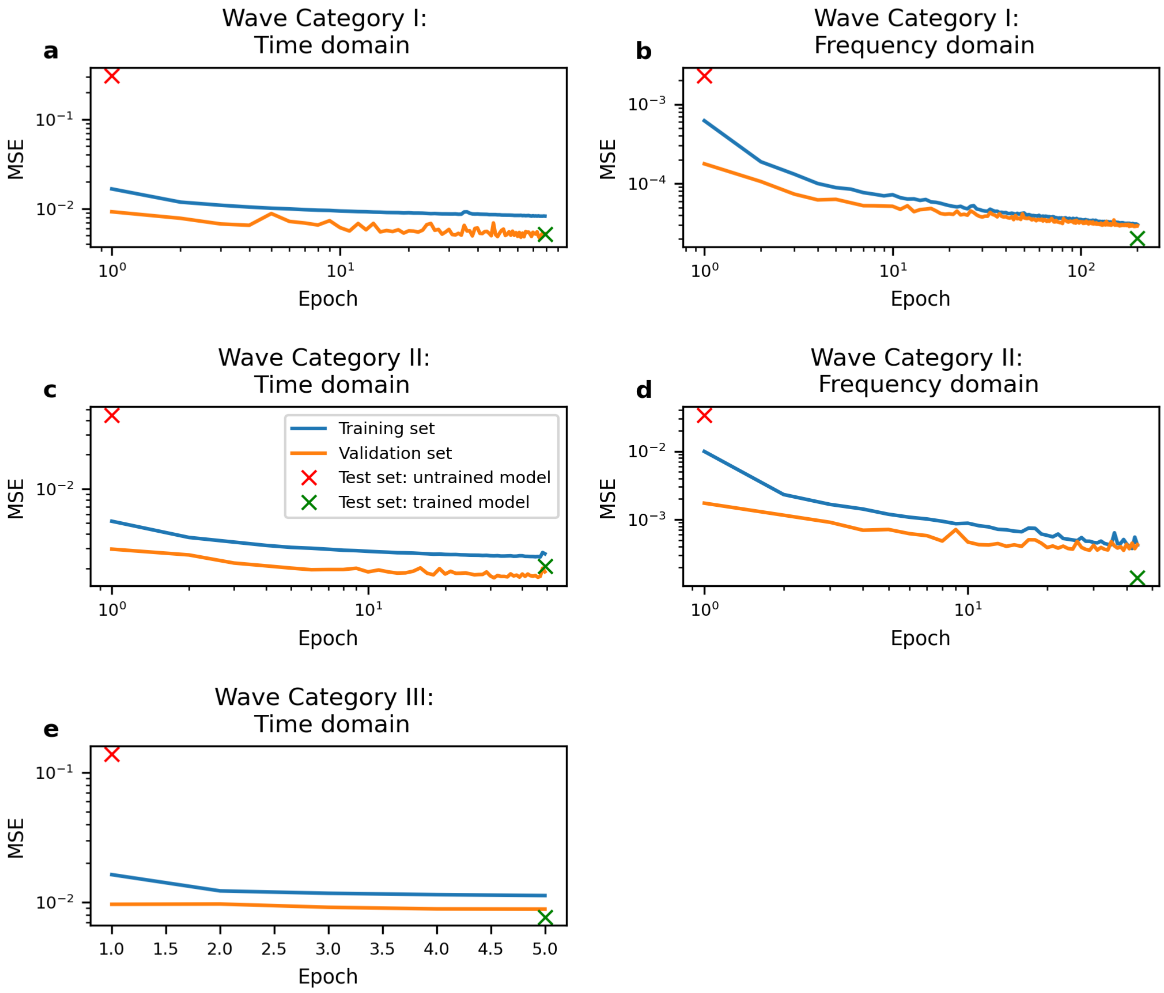

Figure SI 7.1: Training history of the RNNs for **a)** Wave Category I: Time domain RNN, **b)** Wave Category I: Frequency domain RNN,**c)** Wave Category II: Time domain RNN, **d)** Wave Category II: Frequency domain RNN, **e)** Wave Category III: Time domain RNN. Mean squared error (MSE) of the training set (*blue line*), validation set (*orange line*). The MSE of the untrained model on the test set is indicated by the red cross. The MSE of the test set after the training is complete is indicated by the green cross. Note that due to the randomly selected starting point and propagation length given to the experiments in the test set, the value of the MSE can vary slightly.

References

[1] A. V. Babanin, "On a wave-induced turbulence and a wave-mixed upper ocean layer," *Geophysical Research Letters*, vol. 33, no. 20, pp. 1–6, 2006.

[2] Y. Kato and M. Oikawa, "Wave Number Downshift in Modulated Wavetrain trough a Nonlinear Damping Effect," *Journal of the Physical Society of Japan*, vol. 64, no. 12, pp. 4660–4669, 1995.
